# The structure of *Plasmodium falciparum* serine hydroxymethyltransferase reveals a novel redox switch that regulates its activities

**DOI:** 10.1107/S1399004714005598

**Published:** 2014-05-23

**Authors:** Penchit Chitnumsub, Wanwipa Ittarat, Aritsara Jaruwat, Krittikar Noytanom, Watcharee Amornwatcharapong, Wichai Pornthanakasem, Pimchai Chaiyen, Yongyuth Yuthavong, Ubolsree Leartsakulpanich

**Affiliations:** a National Center for Genetic Engineering and Biotechnology, 113 Thailand Science Park, Paholyothin Road, Klong 1, Klong Luang, Pathumthani 12120, Thailand; b Department of Biochemistry and Center for Excellence in Protein Structure and Function, Faculty of Science, Mahidol University, Bangkok, Thailand

**Keywords:** *Plasmodium falciparum*, serine hydroxymethyltransferase, antimalarial target, protein engineering, disulfide/sulfhydryl switch

## Abstract

*Plasmodium falciparum* serine hydroxymethyltransferase (*Pf*SHMT), an enzyme in the dTMP synthesis cycle, is an antimalarial target because inhibition of its expression or function has been shown to be lethal to the parasite. As the wild-type enzyme could not be crystallized, protein engineering of residues on the surface was carried out. The surface-engineered mutant *Pf*SHMT-F292E was successfully crystallized and its structure was determined at 3 Å resolution. The *Pf*SHMT-F292E structure is a good representation of *Pf*SHMT as this variant revealed biochemical properties similar to those of the wild type. Although the overall structure of *Pf*SHMT is similar to those of other SHMTs, unique features including the presence of two loops and a distinctive cysteine pair formed by Cys125 and Cys364 in the tetrahydrofolate (THF) substrate binding pocket were identified. These structural characteristics have never been reported in other SHMTs. Biochemical characterization and mutation analysis of these two residues confirm that they act as a disulfide/sulfhydryl switch to regulate the THF-dependent catalytic function of the enzyme. This redox switch is not present in the human enzyme, in which the cysteine pair is absent. The data reported here can be further exploited as a new strategy to specifically disrupt the activity of the parasite enzyme without interfering with the function of the human enzyme.

## Abstract 2

The crystal structure of *P. falciparum* SHMT revealed snapshots of an intriguing disulfide/sulfhydryl switch controlling the functional activity.

## 1. Introduction   

In spite of the great effort dedicated to the control of malaria, the disease remains a major public health threat in several tropical and subtropical countries (World Health Organization, 2012 ▶). Chemotherapy is presently the only effective method available to treat infected patients. However, the emergence of widespread drug resistance has rendered many commonly deployed drugs ineffective. Therefore, novel antimalarial drugs targeting proteins outside the realm of previously validated targets are needed.

Serine hydroxymethyltransferase (SHMT), a ubiquitous pyridoxal-5′-phosphate (PLP)-dependent enzyme that catalyzes the interconversion of serine and tetrahydrofolate (THF) to glycine and 5,10-methylenetetrahydrofolate (MTHF), is one of the three enzymes in the dTMP cycle. The pathway not only provides dTMP for DNA synthesis, but is also involved in recycling different forms of reduced folates used for the synthesis of purines, choline and other amino acids (Müller & Hyde, 2013 ▶). Unlike the human host, *Plasmodium* parasites rely mainly on the *de novo* synthesis of dTMP, making the dTMP cycle a good target for the development of antimalarial drugs. Additionally, the expression of SHMT is enhanced remarkably during the cell-multiplication stage (Nirmalan *et al.*, 2002 ▶; Pornthanakasem *et al.*, 2012 ▶). Gene targeting experiments have demonstrated that *Plasmodium* SHMT is indispensable for the growth and development of parasites (Pornthanakasem *et al.*, 2012 ▶). These facts signify the vital role of this enzyme as a therapeutic target. The biochemical properties of malarial SHMTs are distinct from those of other SHMTs (Maenpuen *et al.*, 2009 ▶; Sopitthummakhun *et al.*, 2009 ▶, 2012 ▶), and antifolates that were previously developed for dihydrofolate reductase and thymidylate synthase inhibit SHMT at micromolar levels (Pang *et al.*, 2009 ▶; Sopitthummakhun *et al.*, 2012 ▶). In addition, the ligand binding environment and inactivation kinetics using thiosemicarbazide have recently been found to differ between malarial SHMT and human SHMT (*h*SHMT) (Pinthong *et al.*, submitted). Therefore, there should be a great opportunity to design and develop new families of effective antimalarial drugs.

In order to use malarial SHMT as a target for the design of effective inhibitors, crystal structures of the enzyme are needed. Here, we report the successful crystallization of surface-engineered *Pf*SHMT variants in which the enzyme activity was not perturbed. The crystal structure of *Pf*SHMT determined at 3 Å resolution reveals the presence of a unique cysteine pair that acts as a switch to control the THF-dependent activity of *Pf*SHMT. Reduction of the disulfide by the addition of dithiothreitol activates the enzyme. The redox switch discovered here offers a new strategy for the design and development of new selective drugs against malaria.

## 2. Materials and methods   

### 2.1. Protein engineering and expression of *Pf*SHMTs   

pET100/D-*Pf*SHMT previously constructed for the expression of N-terminal His_6_-tagged *Pf*SHMT (Maenpuen *et al.*, 2009 ▶) was used as a template for site-directed mutagenesis. Mutation sites were predicted based on a *Pf*SHMT model constructed from *Escherichia coli* SHMT (*Ec*SHMT; PDB entry 1dfo; Scarsdale *et al.*, 2000 ▶) using *ESyPred*3*D* (Lambert *et al.*, 2002 ▶). Site-directed mutagenesis for surface-engineered mutants (F135A, F292E, L302K and F389K) and those involved in disulfide-bridge formation (C125P and C364A/S) was performed using the QuikChange site-directed mutagenesis kit (Stratagene, California, USA). All expression constructs were sequenced at 1st BASE (Selangor Darul Ehsan, Malaysia) to verify the sequence.

The expression of soluble wild-type *Pf*SHMT and variants was carried out in *E. coli* BL21-CodonPlus^®^ (DE3)-RIL as described previously (Maenpuen *et al.*, 2009 ▶). Briefly, *E. coli* BL21-CodonPlus (DE3)-RIL cells harbouring each expression plasmid of *Pf*SHMT were cultured in Luria–Bertani (LB) medium containing 50 µg ml^−1^ ampicillin and 34 µg ml^−1^ chloramphenicol at 37°C until the OD_600_ reached 0.8. The expression of soluble *Pf*SHMT was then induced with 0.4 m*M* IPTG at 20°C for 22 h. The cells were harvested by centrifugation at 4420*g* for 10 min and stored at −20°C.

### 2.2. Protein purification   

Cells expressing *Pf*SHMTs were suspended in binding buffer [100 m*M* potassium phosphate buffer pH 7.2, 500 m*M* KCl, 100 m*M* imidazole, 10%(*v*/*v*) glycerol] before being French-pressed at 10.3 MPa twice. The supernatant was separated from cell debris by centrifugation at 20 000*g* for 1–2 h before the addition of 30 µ*M* PLP (in water) to the crude extract. PLP was added to prevent loss of cofactor and to ensure maximum formation of the holoenzyme. The crude extract was gently stirred and loaded onto an Ni-Sepharose column (20 ml; 2.5 cm diameter × 4 cm). The impurities were washed out using 100 ml binding buffer and the expected protein was eluted stepwise using elution buffers I [100 m*M* potassium phosphate buffer pH 7.2, 500 m*M* KCl, 150 m*M* imidazole, 10%(*v*/*v*) glycerol], II (buffer I with 100 m*M* KCl and 200 m*M* imidazole) and III (buffer I with 100 m*M* KCl and 300 m*M* imidazole). Fractions of 9 ml were collected and 30 m*M* β-mercaptoethanol was added to prevent disulfide-bond formation. Fractions containing *Pf*SHMT were identified using 12% SDS–PAGE and were combined and concentrated using Millipore centrifugal concentrators of 30 kDa molecular-weight cutoff and buffer-exchanged with 100 m*M* Tris–HCl pH 7, 100 m*M* KCl, 10 m*M* dithiothreitol, 20%(*v*/*v*) glycerol. After reducing the imidazole concentration to lower than 0.1 µ*M*, the protein was filtered through a 0.22 µm Millipore Eppendorf tube by centrifugation at 18 000*g* for 5 min to discard the unwanted particles. The protein was divided into small portions of 0.2 ml each and stored at −80°C. The concentration of the protein was evaluated using the Bradford technique (Bradford, 1976 ▶) using BSA as a standard. The purity of the purified protein was confirmed by SDS–PAGE.

### 2.3. Spectroscopic measurements   

UV–visible absorption spectra of the enzymes were recorded with an Agilent 8453 diode-array spectrophotometer (Hewlett-Packard, Palo Alto, California, USA). All spectra were recorded at 25°C.

### 2.4. Enzyme assays   

The THF-dependent activity of SHMT was measured using a coupling assay with SHMT-methylenetetrahydrofolate dehydrogenase (MTHFD) as described previously (Sopitthummakhun *et al.*, 2012 ▶). The reaction mixture consisted of 2 m*M* l-serine, 0.4 m*M* THF, 0.25 m*M* NADP^+^, 5 µ*M* MTHFD and *Pf*SHMT. The increase in absorbance at 375 nm was monitored (∊_375_ = 1.92 m*M* ^−1^ cm^−1^). To determine the *K*_m_ of l-serine, the concentration of THF was maintained at 0.4 m*M* and l-serine was varied in the range 0.125–4.0 m*M*. To determine the *K*_m_ of THF, the concentration of l-serine was fixed at 2 m*M* and that of THF was varied in the range 0.0125–0.8 m*M*. The *K*_m_^app^ and *k*_cat_^app^ values were calculated using nonlinear least-square algorithms in *Kaleidagraph* (Synergy Software, Reading, Pennsylvania, USA).

For the dithiothreitol-dependent study, the *Pf*SHMT activity was assayed in a system containing 50 m*M* HEPES pH 7.4 and 0.5 m*M* EDTA with 1 m*M* dithiothreitol either omitted or included. A similar procedure was used for *h*SHMT activity determination, except that the pH was 7 and the MTHFD concentration was 10 µ*M* (Pinthong *et al.*, submitted).

### 2.5. Protein crystallization   

*Pf*SHMT was crystallized using the microbatch method (Chitnumsub *et al.*, 2004 ▶; D’Arcy *et al.*, 1996 ▶; Chayen, 1996 ▶) in a 60-well plate (1 mm diameter at the bottom of each well) covered with 6 ml baby oil (Cussons; a mixture of mineral oil, olive oil and vitamin E; PZ Cussons, Thailand). Protein–ligand complexes were prepared by mixing 60 µl purified His_6_-tagged *Pf*SHMT protein (15–18 mg ml^−1^) with 6 µl each of the following solutions: 20 m*M* l-serine, 20 m*M* PLP and 20 m*M* folinic acid. The mixture was equilibrated on ice for 30 min to allow complete complex formation. Crystallization was set up on a microplate by first pipetting 1 µl of the protein complex into the well layered with oil followed by 1 µl crystallization solution without touching the protein drop. Crystals of the *Pf*SHMT F292E or F389K mutants were grown at 293 K in 27–30%(*v*/*v*) PEG 4000, 0.30–0.32 *M* MgCl_2_ and 0.1 *M* either MES buffer pH 6.5 or HEPES pH 7.0.

### 2.6. Data collection, structure determination and refinement   

A single crystal was flash-vitrified in liquid nitrogen using Fomblin^®^ Y as a cryoprotectant. X-ray diffraction data were collected at 100 K at a wavelength of 1 Å using an ADSC Quantum 315 CCD detector on beamline 13B1, NSRRC, Taiwan. Data were processed using the *HKL*-2000 package (Otwinowski & Minor, 1997 ▶). X-ray diffraction data and refinement statistics are listed in Table 1 ▶. The structure of *Pf*SHMT was determined by molecular replacement using *AMoRe* (Navaza, 1994 ▶) in the *CCP*4 suite (Winn *et al.*, 2011 ▶) with a chain *A* protomer from the *Ec*SHMT coordinates (PDB entry 1dfo; 41% sequence identity to *Pf*SHMT; Scarsdale *et al.*, 2000 ▶) as the template. Rotation and translation solutions of each of the four molecules per asymmetric unit were obtained by sequentially performing a translation search with fixed and refined known solutions. The top solutions containing two close contacts have an *R* factor of 49.3%, a Corr_*I* of 43.5%, a Corr_*F* of 36.1% and 52.7% solvent content. Model building and structure refinement were carried out using *Coot* (Emsley & Cowtan, 2004 ▶; Emsley *et al.*, 2010 ▶) and *REFMAC*5 (Murshudov *et al.*, 2011 ▶). The structure was validated in *PROCHECK* (Laskowski *et al.*, 1993 ▶). Superposition of structures was carried out using *LSQMAN* (Kleywegt, 1996 ▶) in *Coot*.

### 2.7. Protein Data Bank code   

Atomic coordinates and structure factors have been deposited with PDB code 4o6z.

## 3. Results and discussion   

### 3.1. Surface engineering and crystallization of *Pf*SHMT   

Crystallization of *Pf*SHMT remains a great challenge and has been unsuccessful despite the fact that more than 1000 conditions were screened. In this study, a rational surface-engineering approach was pursued to enhance the quality of crystal packing in crystallization. This approach has been used successfully in other studies to improve protein crystallization (Evdokimov *et al.*, 2008 ▶). A homology model of *Pf*SHMT was constructed based on the *Ec*SHMT structure (PDB entry 1dfo; 41% identity). Four solvent-exposed hydrophobic and one positively charged amino acids were identified and mutated, namely F135A/G, F292E, L302K, F389K and K396E (Supplementary Fig. S1^1^). These mutations were designed to enhance protein solubility, leading to more facile crystallization without functional perturbation of the enzyme. Besides, these mutations are at sites distant from the active site and are not expected to significantly affect the enzyme properties (Supplementary Fig. S1).

The surface-engineered *Pf*SHMT variants (15–18 mg ml^−1^) were co-crystallized with l-serine, PLP and folinic acid. Two of the five variants, F292E and F389K, readily yielded protein crystals of suitable size and diffraction quality in a buffer comprising 0.1 *M* MES pH 6.5, 0.2 *M* MgCl_2_, 25%(*v*/*v*) PEG 4000 from the JBScreen Classic 2 kit (Jena Bioscience), while other variants gave many microcrystals in a hexagonal form, except for K396E which yielded no crystals. Crystal optimization was performed with various concentrations of MgCl_2_ and PEG 4000, as well as various buffers in the pH range 6.5–9.0. *Pf*SHMTs with F292E and F389K mutations yielded hexagonal crystals with dimensions of 0.15 × 0.06 × 0.15 mm in 4 d (Supplementary Fig. S2). Crystals of *Pf*SHMT-F292E diffracted to 3 Å resolution and those of *Pf*SHMT-F389K diffracted to less than 3 Å resolution. The crystals belonged to space group *P*6_1_, with unit-cell parameters *a* = 254.95, *b* = 254.95, *c* = 61.40 Å, α, β = 90, γ = 120°. The Matthews coefficient of the crystal is 2.6 Å^3^ Da^−1^, corresponding to four protomers per asymmetric unit. Data-collection statistics and structure-refinement parameters are given in Table 1 ▶. The kinetic properties of *Pf*SHMT-F292E were examined to measure the effects of the introduced mutation. The *Pf*SHMT-F292E variant exhibited similar kinetic properties to those of the wild-type protein (Table 2 ▶), indicating that the change to a hydrophilic residue only affected the surface properties and did not have a significant effect on the catalytic activity of the enzyme. Therefore, it could be concluded that this variant is representative of the wild-type protein.

### 3.2. Crystal structure of *Pf*SHMT   

#### 3.2.1. Overall structure   

**Figure 1:**
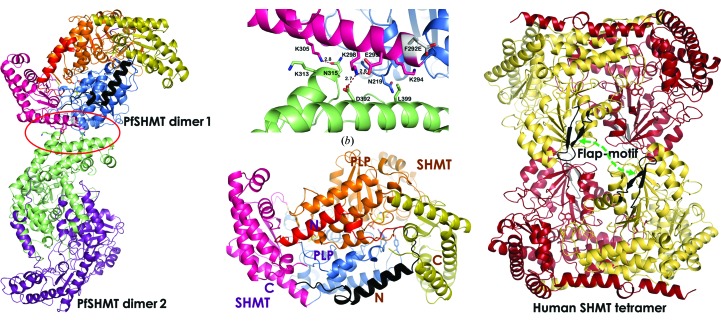
Crystal structures of *Pf*SHMT and *h*SHMT. (*a*) Two homodimers of the *Pf*SHMT-F292E structure at 3 Å resolution, showing two protomers in dimer 1 and dimer 2. (*b*) *Pf*SHMT dimer interface in crystal packing. (*c*) The three domains of the *Pf*SHMT protomer: N (residues 1–34), large PLP binding (residues 35–290) and C domains (residues 291–442); PLP is shown in yellow stick represenation. (*d*) The native tetrameric form of *h*SHMT shown in brown and yellow with the flap motif (residues 274–285) in black.

The structure of *Pf*SHMT was determined at 3 Å resolution in space group *P*6_1_ with four protomers per asymmetric unit. Unlike the human SHMT (*h*SHMT) tetramer, the four protomers of *Pf*SHMT exist as two individual homodimers (Fig. 1 ▶ *a*), with only a few interactions between the homodimers. Two pairs of hydrogen bonds from residues on each C-terminal domain, namely Lys298–Asp′392 and Lys305–Asn′315, are found to hold the two homodimers in an asymmetric unit (Fig. 1 ▶ *b*). Each protomer of *Pf*SHMT homodimer can be divided into three domains, namely the N-terminal domain, the PLP/substrate binding domain and the C-terminal domain (Fig. 1 ▶ *c*). The N-terminal domain, ranging from residues 1 to 34, lies on the top of the PLP pocket. The PLP/substrate binding domain, which is the largest of the three domains, consisting of residues 35–290, covers PLP, the amino-acid substrate and the pterin moiety of the THF cofactor binding pockets. PLP binds to the pockets formed by the two PLP/substrate domains, linking the two subunits of the *Pf*SHMT homodimer (see §[Sec sec3.2.2]3.2.2). The C-terminal domain (residues 291–442) of each *Pf*SHMT protomer, in combination with the PLP binding domain, forms the THF binding pocket.

#### 3.2.2. PLP binding sites   

**Figure 2:**
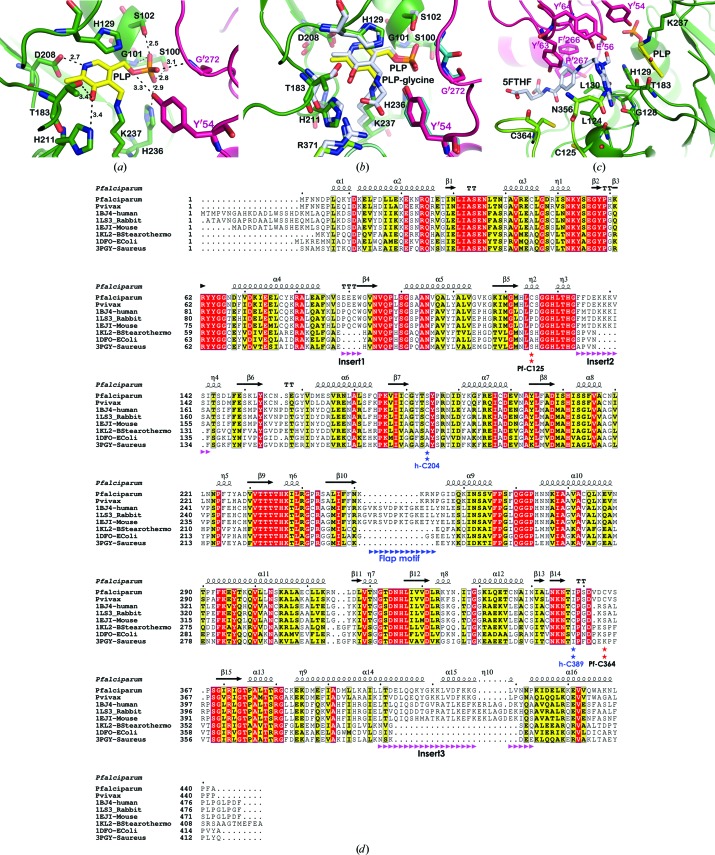
(*a*) PLP binding interaction in the *Pf*SHMT homodimer with PLP in yellow, amino-acid residues from one *Pf*SHMT protomer in green and those from the other protomer in pink. Dashed lines indicate hydrogen-bond distances (in Å). Residues Ser100, Ser102, His129, Thr183, Asp208, His211, His236 and Lys237 are from one protomer and Tyr′54 and Gly′272 are from the other protomer. (*b*) PLP–glycine binding pocket and (*c*) THF binding pocket with bound 5FTHF from *Ec*SHMT from superimposition of *Pf*SHMT and *Ec*SHMT (PDB entry 1dfo) to map the substrate binding pockets and residues at the pockets. (*d*) Primary amino-acid sequence alignment of SHMTs indicates three inserted sequences in *Pf*SHMT and mammalian SHMTs compared with bacterial SHMTs. The *Pf*SHMT residues Cys125 and Cys364 and *h*SHMT residues Cys204 and Cys389 as well as the human flap motif are indicated.

The two PLP and substrate binding pockets of the dimeric *Pf*SHMT are constituted by residues from both protomers (Fig. 1 ▶ *c*). The PLP binding pocket is composed of Ser100, Gly101, Ser102, His129, Thr183, Asp208, His211, His236 and Lys237 from one subunit and Tyr′54, Glu′56, Tyr′64 and Gly′272 from the neighbouring subunit. The pyridoxal ring binds deeply in the pocket, with the C4A aldehyde of the pyridine ring forming a Schiff-base linkage to Lys237 N^∊^. The protonated N1 of the pyridine ring forms a hydrogen bond to Asp208 O^δ1^. O3 of the PLP forms a hydrogen bond to His211 N^η^. Moreover, the pyridine ring is stabilized by a π–π interaction with His129. The THF-dependent activity of *Pf*SHMT-H129A was only 3% of the specific activity of the wild-type *Pf*SHMT (0.1 *versus* 3.71 µmol min^−1^ mg^−1^). The phosphate of the PLP Schiff base forms hydrogen bonds to Ser100, Ser102, His236, Tyr′54 and Gly′272 (Fig. 2 ▶ *a*). The rest of the PLP is stabilized by van der Waals interactions from hydrophobic residues around the site. Therefore, Schiff-base formation not only activates the C4A carbonyl but also secures the PLP in a proper geometry at the active site.

#### 3.2.3. Substrate binding sites   

Although the crystals of *Pf*SHMT were grown from a solution of the enzyme in the presence of PLP and l-serine, the latter was not observed in the structure, likely owing to its high dissociation constant (Maenpuen *et al.*, 2009 ▶). Therefore, in order to determine the residues involved in the binding of l-serine and THF, the *Pf*SHMT structure was superimposed with the *Ec*SHMT ternary complex containing the PLP–glycine Schiff base and 5-formyltetrahydrofolate (5FTHF; Fig. 2 ▶ *b*). The amino-acid binding pocket is well conserved, while differences are observed at the THF binding site. Similarly to *Ec*SHMT, PLP–glycine Schiff-base binding in *Pf*SHMT involves interaction of Ser34, Tyr′54, Tyr′64, His129, Thr183, Asp208, His211, Thr234, His236, Gly′272 and Arg371 (Fig. 2 ▶ *b*). THF binding inter­actions could be divided into two parts: firstly binding of the pterin ring and secondly binding of the *p*-aminobenzoate (*p*ABA) and l-glutamate moieties. The conserved polar interaction at the pterin moiety of THF, which is well recognizable among various organisms, includes hydrogen bonds from the main chain of Leu124, Gly128 and Leu130 and the carbonyl side chain of Asn356 based upon the 5FTHF interaction in *Ec*SHMT (Fig. 2 ▶ *c*). The *p*ABA ring of THF is stabilized *via* the conserved π–π interaction of Tyr′63 and Phe′266, yet the pocket geometry from this part towards the l-glutamate group is distinguishable owing to the structural compactness of the SHMT homodimer and loop movement (see §[Sec sec3.2.4]3.2.4). The binding feature of THF illustrated here is based on the binding of 5FTHF in *Ec*SHMT which may be slightly different from that of THF, which contains no substituent at the N5 position of the pterin ring.

It has been reported that the flexibility of the folate pocket is in general much greater than that of the amino-acid pocket (Rao & Rao, 1980 ▶; Trivedi *et al.*, 2002 ▶; Szebenyi *et al.*, 2000 ▶). Limited space for amino-acid accommodation may have a direct impact on amino-acid affinity, selectivity and reactivity. Previous experiments with *Pf*SHMT indicate the occurrence of different intermediates upon incubation of the enzyme with different amino acids (Maenpuen *et al.*, 2009 ▶). With d-serine and glycine substrates, a quinonoid intermediate was formed, while aldimine formation was observed in the case of l-serine.

#### 3.2.4. Prerequisite of the sulfhydryl state for THF binding   

**Figure 3:**
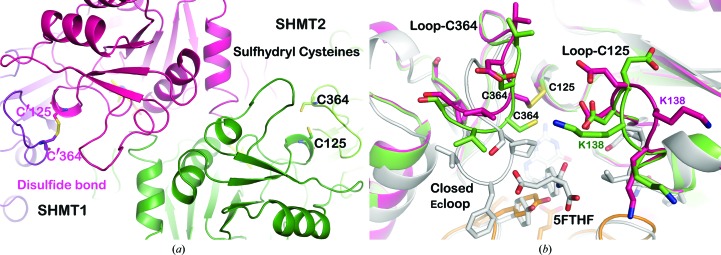
Cysteine pair at the THF binding pocket. (*a*) Conformation of loop-Cys125 and loop-Cys364 at the THF binding pocket of *Pf*SHMT, controlled by the reduction state of the cysteine pair Cys125 and Cys364. The disulfide bond in pink and the corresponding sulfhydryl groups in green of Cys125 and Cys364 control the movement of the surface loop-Cys125 and loop-Cys364 (residues 126–143 and 356–369, respectively) critical for *Pf*SHMT functional activity. (*b*) Superposition of *Pf*SHMT1 (pink), *Pf*SHMT2 (green) and *Ec*SHMT (PDB entry 1dfo; white) showing the THF pocket and bound 5FTHF from the *Ec*SHMT structure. A conformational change of loop-Cys364 towards the THF binding pocket promoted the binding of the THF substrate in the active sulfhydryl enzyme (in green).

In contrast to the other SHMT structures reported to date, the structure of *Pf*SHMT showed the presence of a disulfide bond on the surface loops at the entrance to the THF pocket between Cys125 and Cys364 from the PLP and the C-terminal domains, respectively (Fig. 3 ▶ *a*). This disulfide bond might be reversible as it was observed in only one subunit of the *Pf*SHMT homodimer. The reason for the unequal reduction of the four protomers has yet to be explored. The conformations of these loops may involve different reduction states of the protomers. A comparison of the local conformations of Cys125 and Cys364 in the four protomers showed that the C^α^—C^α^ distances of the oxidized Cys364 from protomer *A* to the reduced states in the other three protomers are about 1.8–2.2 Å, while the Cys125 conformations are nearly identical. Nonetheless, such a disulfide bridge has not been reported in any SHMT studied to date. Moreover, an amino-acid sequence alignment of SHMTs from various organisms shows that only *Plasmodium* species possess the cysteine pair at the THF binding pocket (Fig. 2 ▶ *d*). Examination of the structure suggests that formation of the disulfide bond would interfere with THF anchoring and that the side chains at Cys125 and Cys364 need to be in the reduced state (sulfhydryl form) to give the active form of the enzyme. In *Ec*SHMT, only three cysteines are found and none of them is close to the l-serine or THF binding pockets. The presence of the disulfide bond in *Pf*SHMT prevents the loop movement which is required for THF binding. This structural feature explains the loss of activity in the absence of reducing agent reported previously by our group (Maenpuen *et al.*, 2009 ▶). In human or other eukaryotic SHMTs, there are two cysteine residues, for example Cys204 and Cys389 (equivalent to Ser184 and Ile358, respectively, in *Pf*SHMT), at the THF pocket of *h*SHMT, which are the nearest cysteines to the pair observed in *Pf*SHMT. Residue Cys389 is located on the equivalent loop-Cys364 (residues 356–369) of *Pf*SHMT but at a different position, while Cys204 is on the loop near the *Pf*SHMT loop-Cys125 (residues 126–143) (Fig. 2 ▶ *d*). However, the C^α^—C^α^ distance between Cys204 and Cys389 in *h*SHMT is 9.9 Å, which is longer than the 7.2 Å sulfhydryl C^α^—C^α^ distances of Cys125 and Cys364 in the *Pf*SHMT structure. The longest C^α^—C^α^ distance of the disulfide extracted from protein structures in the PDB is about 6.8 Å (Fass, 2012 ▶). This implies that a similar disulfide bond cannot be formed in *h*SHMT. In order to explore this point further, the effects of dithiothreitol on the activities of *Pf*SHMT and *h*SHMT were studied in order to understand the role of this disulfide/sulfhydryl state in the regulation of SHMT activity (see §[Sec sec3.3]3.3).

#### 3.2.5. Structural differences between *Pf*SHMT, bacterial SHMT and *h*SHMT   

Although the overall structure of *Pf*SHMT shows high similarity to SHMTs from various organisms, differences at the folate binding site and conformational differences at the amino-acid binding pocket may contribute to the difference in enzyme activity and substrate and inhibitor affinities. Unlike *h*SHMT and rabbit SHMT (*r*SHMT), which exist as tetramers (Renwick *et al.*, 1998 ▶), the active form of *Pf*SHMT is a homodimer with a molecular weight of about 85 kDa (Maenpuen *et al.*, 2009 ▶), similar to bacterial enzymes such as *Ec*SHMT and *Bacillus stearo­thermophilus* (*Bs*) SHMT (Scarsdale *et al.*, 2000 ▶; Trivedi *et al.*, 2002 ▶). *Pf*SHMT shares ∼47 and ∼42% sequence identity with mammalian and bacterial SHMTs, respectively. Structurally, a PLP and an amino-acid substrate binding pocket are conserved among SHMTs from different organisms. Superposition of *Pf*SHMT with mammalian and bacterial SHMTs suggested that the homodimer of *Pf*SHMT shares more similar features with mammalian than with bacterial SHMT, in particular at the THF binding site, with an r.m.s.d. of 0.90 Å for 435 C^α^ atoms and 1.36 Å for 397 C^α^ atoms for *h*SHMT and *Ec*SHMT, respectively. Firstly, both *Pf*SHMT and mammalian SHMTs have four-residue inserts (insert 1) from residues 89 to 92 in *Pf*SHMT and 107 to 110 in *h*SHMT, each of which is juxtaposed to the human flap motif (Fig. 2 ▶ *d*). Secondly, *Pf*SHMT contains an extra sequence at residues 134–143 (part of loop-Cys125) forming a longer polar loop with a conserved sequence of FFDEKKKVSI in *Pf*SHMT, equivalent to residues 153–162 and 131–137 in *h*SHMT and *r*SHMT (FMTDKKKISA), respectively. Since the loop is located close to the l-glutamate tail of THF and has sequence KKK, it is likely that interaction with the tail affects binding and catalysis. The structure of *r*SHMT complexed with triGlu-5-formyltetrahydrofolate (H_4_PteGlu_3_; PDB entry 1ls3; Fu *et al.*, 2003 ▶) with a three γ-glutamate tail showed that the Lys135 (equivalent to Lys138 in *Pf*SHMT and Lys157 in *h*SHMT) side chain is hydrogen-bonded to the third γ-glutamate of the tail, and the dissociation constant of H_4_PteGlu_3_ is 14 times lower than that of H_4_PteGlu (Fu *et al.*, 2003 ▶). In contrast to *Pf*SHMT and *h*SHMT, only a short loop (SPVNF) exists in *Ec*SHMT (residues 130–134) and *Bs*SHMT (residues 127–131). This polar loop is important for the increased affinity of polyglutamate forms of THF. In *Ec*SHMT, only a twofold increase in the binding affinity of H_4_PteGlu_*n*_ is observed when the γ-glutamate chain length is varied from 1 to 6, whereas a 20-fold increase is observed for mammalian SHMTs (Fu *et al.*, 2003 ▶). Thirdly, *Pf*SHMT and mammalian SHMTs have an extra helix (at positions 400–423 in *Pf*SHMTand 430–458 in *h*SHMT) introducing an additional helix–loop motif located on the top of the THF binding pocket, close to the l-glutamate group of THF, compared with a short loop in *Ec*SHMT (residues 384–396) and *Bs*SHMT (residues 385–390).

Although the *Pf*SHMT structure resembles *h*SHMT in many aspects, there is a unique β-hairpin structure or so-called flap motif (residues 274–285; KSVDPKTGKEIL) in human/mammalian SHMTs located on the top of the THF binding site (Fig. 1 ▶ *d*) that is absent in *Pf*SHMT. The flap motif in *h*SHMT may be crucial for closing the pocket upon binding of THF so as to increase the catalytic efficiency. Nonetheless, this proposal has not yet been confirmed owing to the unavailability of a THF-bound *h*SHMT structure. In contrast, the human flap motif is replaced by a short loop (residues 251–257) in *Pf*SHMT, making the THF pocket more exposed to the solvent. Moreover, *h*SHMT has a longer N-terminal domain (53 residues) compared with the 34 residues of *Pf*SHMT. The extra N-terminal sequence, residues 13–23 in *h*SHMT, the role of which is still unknown, adopts an α-helical structure, although residues 1–12 were disordered in the crystal structure.

The structure reported here should be useful as a basis for future mechanistic studies to elucidate the reaction mechanism of SHMT. Currently, the mechanism of the THF-dependent SHMT reaction is still under debate as to whether the reaction occurs through retro-aldol cleavage to form free formaldehyde before the formation of MTHF or whether the N5 of THF directly participates in nucleophilic replacement to form the glycine product (Schirch & Szebenyi, 2005 ▶; Schirch, 1998 ▶). The present structural information cannot distinguish between these possibilities because the ternary complex of THF and the external aldimine could not be crystallized. Therefore, the distance between the N5 of THF and the C^β^ atom of l-serine cannot be obtained in order to discriminate between the two mechanisms. It would be useful for the evaluation of any conformational change owing to ligand binding if the structure of such a complex could be obtained in future investigations.

### 3.3. Structure–function relationship: probing the role of Cys125 and Cys364 as a redox switch in *Pf*SHMT catalysis   

**Figure 4:**
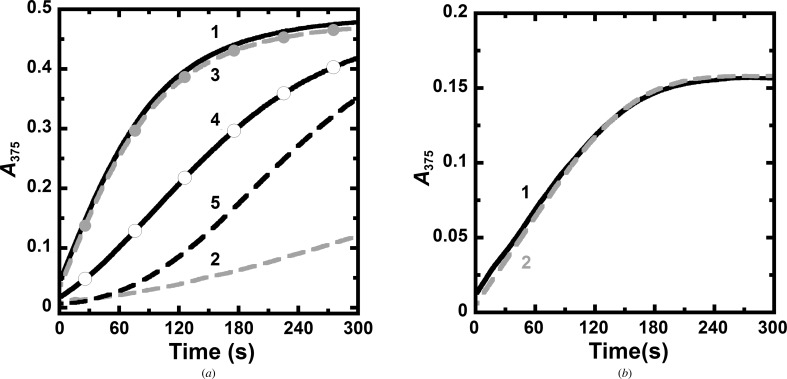
Probing the dithiothreitol-dependent activity of (*a*) *Pf*SHMT and (*b*) *h*SHMT. Proteins were prepared in the presence or absence of dithiothreitol and the assays were conducted in 50 m*M* HEPES pH 7.4 for *Pf*SHMT or pH 7 for *h*SHMT, 0.5 m*M* EDTA omitting or including 1 m*M* dithiothreitol. For *Pf*SHMT, 1 and 2 are activity traces of protein prepared, diluted and assayed in the system with dithiothreitol and without dithiothreitol, respectively, 3 and 4 are the activity traces of protein prepared in the absence of dithiothreitol diluted in buffer containing dithiothreitol for 30 and 5 min, respectively, and assayed in the system with dithiothreitol and 5 is theactivity trace of protein prepared and diluted in the system without dithiothreitol but assayed in the system with dithiothreitol. For *h*SHMT, (1) and (2) are activity traces of protein prepared, diluted and assayed in the system with dithiothreitol and without dithiothreitol, respectively.

Structural analysis of *Pf*SHMT reveals a unique feature: Cys125 and Cys364 are present as a disulfide bond in one subunit and as cysteines with sulfhydryls in another subunit. These residues are situated at the folate binding pocket. It was hypothesized that the cysteine oxidation state might modulate the *Pf*SHMT activity. To test this hypothesis, a reducing agent (dithiothreitol) was used to alter the redox status of these two residues and the catalytic activity of *Pf*SHMT was investigated. Purified *Pf*SHMT was prepared separately in the absence and presence of dithiothreitol, and the activity of each protein preparation was measured in a reaction in which dithiothreitol was either omitted or included. In contrast to the protein prepared and assayed in the presence of dithiothreitol, which yielded the maximum activity, the protein prepared and assayed in the absence of dithiothreitol showed very low or no catalytic activity (Fig. 4 ▶ *a*). Notably, the activity of *Pf*SHMT was gradually regained if the assay condition contained dithiothreitol, or upon pre-incubation of the enzyme in a buffer containing dithiothreitol. A pre-incubation period of 30 min could restore the activity to the level equivalent to that prepared in the presence of reducing agent, and the lag phase was no longer observed (Fig. 4 ▶ *a*).

In the case of *h*SHMT, owing to a longer distance between Cys204 and Cys389 which disfavours disulfide-bridge formation, it is likely that the activity of *h*SHMT is independent of the redox status of these two cysteine residues. Therefore, a similar study as described above for *Pf*SHMT was performed with *h*SHMT. The results indeed confirm the above hypothesis because *h*SHMT prepared in the presence or absence of reducing agent showed similar specific activities (11.5 µmol min^−1^ mg^−1^; Fig. 4 ▶ *b*). It is concluded that *Pf*SHMT and *h*SHMT are different and that in the parasite enzyme the cysteine pair can act as a redox switch by altering the disulfide/sulfhydryl state and the sulfhydryl enzyme is the catalytically active form.

To further test whether the disulfide bond between Cys125 and Cys364 is a redox switch acting in response to dithiothreitol in the activity of *Pf*SHMT, mutations to disrupt the disulfide-bond formation of these residues were carried out. The sequence alignment showed that both Cys125 and Cys346 are nonconserved amino acids (Fig. 2 ▶ *d*). Cys125 is replaced by proline in eukaryotic SHMT, while it is serine or alanine in prokaryotic SHMT. The equivalent position to Cys364 in most organisms is serine, with some exceptions where it is a lysine. Cys125 is situated on a turn of the 3_10_-helix and this helical structure is conserved amongst higher organisms, while Cys364 is on a 14-residue loop. To prevent disulfide-bond formation and to conserve the structural integrity of the flexible loop-Cys346 and the 3_10_-helix of loop-Cys125, C364A/S and C125P variants were constructed and their activities in the assay conditions with and without dithiothreitol were compared. As postulated, the C364A/S variants are functionally active and their activities were independent of dithiothreitol (Table 2 ▶). In general, the kinetic properties of these variants are comparable to those of the wild type. The only exception was the C125P mutant, for which the activity was markedly reduced by more than tenfold (Table 2 ▶). It is possible that in this mutant, other effects on enzymatic activity beside disruption of the disulfide bond also take place.

**Figure 5:**
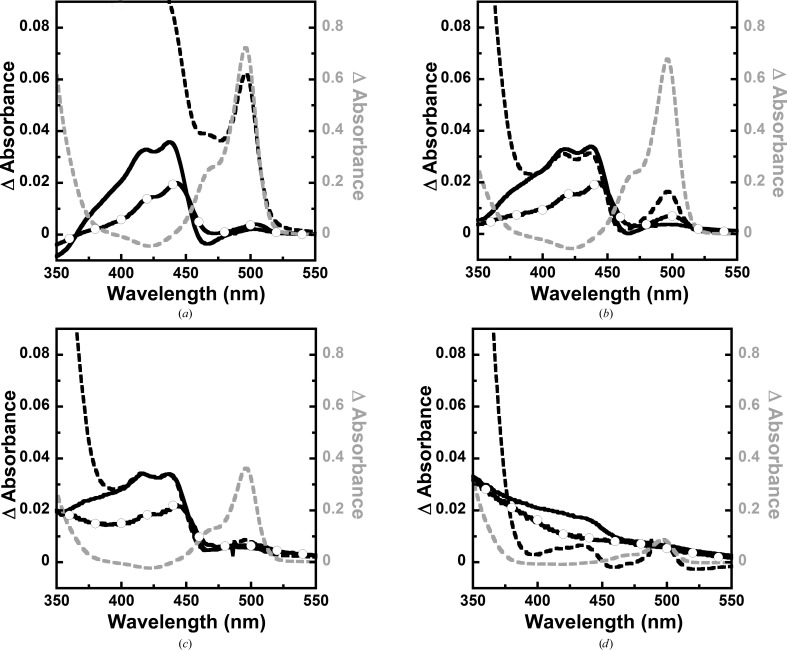
Different spectra for the binding of l-serine (black solid line), l-serine with THF (black dashed line), glycine (black solid line with open circles) and glycine with THF (grey dashed line) to (*a*) wild-type *Pf*SHMT and the (*b*) C364A, (*c*) C364S and (*d*) C125P mutants. The spectra drawn in black use the black scale on the left-hand side and that in grey uses the grey scale on the right-hand side.

These variants were further analyzed for binding to serine or glycine and THF, and changes in the spectra of the PLP intermediates were monitored (Fig. 5 ▶). The spectroscopic properties of PLP-dependent enzymes and the intermediates occurring during catalysis have been well documented and can be used to probe interactions of enzyme-bound PLP with other ligands (Dunathan, 1966 ▶; Eliot & Kirsch, 2004 ▶; Toney, 2005 ▶; Mozzarelli & Bettati, 2006 ▶; Amadasi *et al.*, 2007 ▶; McCoy *et al.*, 2007 ▶). The overall spectral changes for the C364A and C364S mutants were similar to those of the wild type: absorption changes around 410–440 nm for enzyme incubated with serine, 410–440 and 500 nm for enzyme incubated with glycine and a pronounced change at 500 nm on subsequent addition of THF (Figs. 5 ▶ *a*, 5 ▶ *b* and 5 ▶ *c*). It was noted that the C364S mutant displayed a smaller absorbance change compared with the wild type and the C364A mutant. The results indicate that these variants can bind and interact with ligands similar to the wild-type enzyme. Mutation to alanine only disrupted disulfide-bridge formation but did not have any negative effect on enzyme activity, while mutation to serine may slightly perturb the environment or conformation of loop-Cys364, as a higher concentration of THF was required to reach the maximum activity (data not shown). For the C125P mutant, no significant change in the absorbance at 410–440 and 500 nm was noted on incubation with serine or glycine (Fig. 5 ▶ *d*). However, absorbance at around 500 nm for the enzyme-quinonoid intermediate became pronounced when THF was subsequently added, and the signal of the enzyme initially incubated with glycine was higher than that with serine. This implies that the substrate binding properties of the C125P mutant were impaired, leading to low catalytic activity. The effect of this mutation should be further investigated in detail.

## 4. Conclusions   

The reduced efficacy of currently used antimalarial drugs owing to the widespread occurrence of antimalarial-resistant parasites underscores the need for new effective drugs. SHMT is a potential drug target against which new such drugs may be discovered. This study presents the first description of the crystal structure of *Plasmodium* SHMT, an essential enzyme for parasite growth (Pornthanakasem *et al.*, 2012 ▶). The study has identified a novel cysteine pair (Cys125 and Cys364) whose redox status governs *Pf*SHMT activity. These cysteine residues act as a redox switch: the oxidized form (presumably the disulfide form) is deleterious to enzyme activity, while the enzymatic function is regained in the reduced form. The disulfide-bond formation of these two cysteine residues was shown to prevent catalysis, presumably by freezing the loops at the active site essential for THF substrate binding. It was noted that the flexibility around the disulfide bridge lies in loop-Cys364. For *h*SHMT, in which the cysteine pair is absent, the enzymatic activity is independent of the disulfide/sulfhydryl status, suggesting that the redox switch of the cysteine pair is unique to *Plasmodium* species. It should be explored in future studies whether the parasite uses this redox-switch mechanism to control the function of SHMT.

Redox-controlled proteins with diverse functions have been reported (Ryu, 2012 ▶; Reddie & Carroll, 2008 ▶). Examples include OxyR, an *E. coli* transcription factor responsive to oxidative stress (Toledano *et al.*, 1994 ▶; Zheng *et al.*, 1998 ▶; Choi *et al.*, 2001 ▶), INAD, a *Drosophila* scaffold protein for the association of visual signal transduction components in photoreceptors (Liu *et al.*, 2011 ▶), and angiotensinogen, a precursor protein controlling blood pressure (Zhou *et al.*, 2010 ▶). In addition to control of *Pf*SHMT function by alternating between the active and inactive forms of *Pf*SHMT, the redox cycling of *Pf*SHMT may have a translational auto-regulatory role. It has been shown previously that *h*SHMT and *Pf*SHMT could bind their cognate RNAs under reducing conditions, but only the binding of *h*SHMT to its own RNA inhibited translation (Pang *et al.*, 2009 ▶; Liu *et al.*, 2000 ▶). As *Pf*SHMT possesses two redox states, it is possible that each would have distinct binding affinities and that only the stronger binding pair leads to translational auto-regulation of *Pf*SHMT. In the future, comparative studies of gel-mobility shift and *in vitro* translation assays under oxidizing and reducing conditions are needed to provide insights into the role of the *Pf*SHMT redox sensor. Moreover, studies involving proteins controlling redox metabolic balance in parasites such as glutaredoxin, thioredoxin and glutathione reductase should be explored, so that a comprehensive understanding of the metabolic control of folate metabolism by redox balance can be achieved. The results reported here imply that the cysteine redox switch may be another point for the design of inhibitors against malarial parasites since the reduced sulfhydryl cysteines are critical for function. Additionally, some oxidant inhibitors that inhibit the parasite through other mechanisms may exert an extra effect through simultaneous inhibition of parasite SHMT activity. Taken together, the study supports *Plasmodium* SHMT as a new candidate target for antimalarial drug discovery and development.

## Supplementary Material

Supporting Information containing the surface-engineering approaches for the crystallization of PfSHMT and figures showing the mutated residue positions on the PfSHMT homology model and a crystal picture. . DOI: 10.1107/S1399004714005598/mh5125sup1.pdf

PDB reference: serine hydroxymethyltransferase, 4o6z

## Figures and Tables

**Table 1:** Data-collection and refinement statistics for the *Pf*SHMT crystal Values in parentheses are for the highest resolution shell.

Wavelength (Å)	1
Space group	*P*6_1_
Molecules per asymmetric unit	4
Unit-cell parameters (Å, °)	*a* = 254.95, *b* = 254.95, *c* = 61.40, α = β = 90, γ = 120
Resolution (Å)	29.85–2.98 (3.09–2.98)
No. of measured reflections	380143
No. of unique reflections	47265
Multiplicity	8.0 (7.9)
Completeness (%)	99.9 (99.2)
〈*I*/σ(*I*)〉	32.6 (10.2)
*R*_merge_ (%)†	5.2 (20.6)
Wilson *B* factor (Å^2^)	75
Matthews coefficient (Å^3^ Da^−1^)	2.6
Solvent content (%)	52.70
*R* factor/*R*_free_ (%)	22.37/26.96
FOM	0.802
R.m.s.d., bonds (Å)	0.0108
R.m.s.d., angles (°)	1.5357
Ramachandran plot	
Most favoured regions (%)	90.4
Additional allowed regions (%)	9.1
Generously allowed regions (%)	0.6
Disallowed regions (%)	0.0
No. of atoms	
Protein	14066
Water	138
PLP	45
PO4 (phosphate)	5
PDB code	4o6z

† *R*_merge_ = 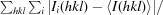 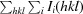, where *I*_*i*_(*hkl*) is the intensity of the *i*th measurement of an equivalent reflection with indices *hkl* and 〈*I*(*hkl*)〉 is the mean intensity of *I*_*i*_(*hkl*) for all *i* measurements.

**Table 2:** Kinetic parameters of *Pf*SHMT variants The *K*_m_ of the C125P variant was not determined (ND) owing to low catalytic activity.

	*K*_m_ (m*M*)		
Protein	L-Serine	THF	*k*_cat_ (s^−1^)
WT	0.123 ± 0.019	0.086 ± 0.005	3.71 ± 0.04
F292E	0.112 ± 0.004	0.077 ± 0.002	3.61 ± 0.16
C125P + dithiothreitol	ND	ND	0.32 ± 0.14
C125P − dithiothreitol	ND	ND	0.24 ± 0.06
C364A + dithiothreitol	0.067 ± 0.008	0.028 ± 0.002	4.00 ± 0.01
C364A − dithiothreitol	0.070 ± 0.008	0.038 ± 0.006	3.91 ± 0.15
C364S + dithiothreitol	0.074 ± 0.007	0.150 ± 0.021	3.77 ± 0.08
C364S − dithiothreitol	0.082 ± 0.019	0.135 ± 0.019	3.84 ± 0.28

## Footnotes

1 Supporting information has been deposited in the IUCr electronic archive (Reference: MH5125).
