# Supplementary material for: The structure of Plasmodium falciparum serine hydroxymethyltransferase reveals a novel redox switch that regulates its activities
Source: Acta Crystallogr D Biol Crystallogr. 2014 May 23;70(Pt 6):1517–27. doi: 10.1107/S1399004714005598 (PMC4051499; doi:10.1107/S1399004714005598)

Supplementary materials

Surface engineering approaches for crystallization of PfSHMT

Based on the homology model generated, F135 is a surface residue on an insert loop $F^{134}FDEKKKVS^{142}$ of PfSHMT, equivalent to $S^{131}PVN^{134}$ loop of EcSHMT (Fig S1). Mutation to a less hydrophobic amino acid G or A was expected to reduce its unfavorable interactions with the polar environment. L302 is situated on a solvent exposed helix, and mutation to K would result in favourable interactions with E384 in the vicinity. F292, located at the end of a solvent-exposed helix with its side chain projecting out to the solvent, was mutated to E to be similar to an equivalent solvent-exposed side chain E283 of EcSHMT. F389 was mutated to a basic amino acid K, so as to introduce hydrogen bonding with E435 or another surrounding acidic amino acid. Finally, K396 was mutated to E to introduce interactions with R314 and K424. These mutations have been made at sites distant from the active site, and are not expected to affect the enzyme properties significantly.

Figure S1 A homodimer of PfSHMT homology model built from ESyPred3D using Ec SHMT, pdb code 1DFO, as a template. Residues (F135, F292, L302 and F389 in ball-stick representation) were chosen for protein engineering.

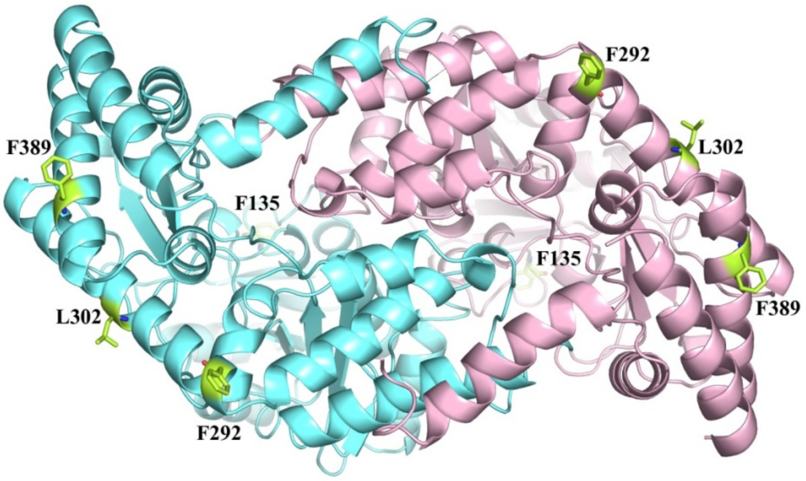

Figure S2 Crystals of PfSHMT-F292E. Crystals were grown in 27% v/v PEG4000, 0.1 M MES buffer pH 6.5 and 0.30-0.32 M $MgCl_2$ at 293K to the size of 0.15x0.06x0.15 mm in 4 days.

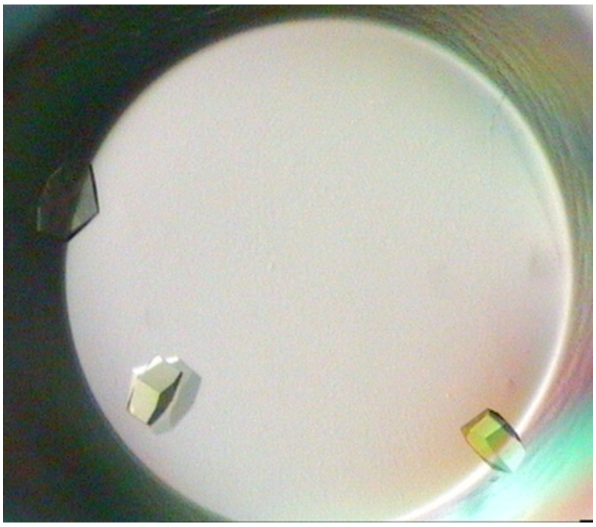

Supplement: Supplementary file 1 [file d-70-01517-sup1.pdf]
